# The HIV treatment cascade and care continuum: updates, goals, and recommendations for the future

**DOI:** 10.1186/s12981-016-0120-0

**Published:** 2016-11-08

**Authors:** Emma Sophia Kay, D. Scott Batey, Michael J. Mugavero

**Affiliations:** 1 School of Social Work, University of Alabama, Box 870314, Tuscaloosa, AL 35487-0314 USA; 2 Department of Social Work, University of Alabama at Birmingham, HB 302F, 1720 2nd Ave South, Birmingham, AL 35294-1260 USA; 3 Department of Medicine, University of Alabama at Birmingham, BBRB 206H, 1720 2nd Ave South, Birmingham, AL 35294-2170 USA

**Keywords:** HIV continuum, HIV treatment cascade, Diagnosis, Retention, Linkage to care, Adherence, Viral suppression

## Abstract

The HIV care continuum is a framework that models the dynamic stages of HIV care. The continuum consists of five main steps, which, at the population level, are depicted cross-sectionally as the HIV treatment cascade. These steps include diagnosis, linkage to care (LTC), retention in care (RiC), adherence to antiretroviral therapy (ART), and viral suppression. Although the HIV treatment cascade is represented as a linear, unidirectional framework, persons living with HIV (PLWH) often experience the care continuum in a less streamlined fashion, skip steps altogether, or even exit the continuum for a period of time and regress to an earlier stage. The proportion of PLWH decreases at each successive step of the cascade, beginning with an estimated 86% who are diagnosed and dropping dramatically to approximately 30% of PLWH who are virally suppressed in the United States (US). In this current issues review, we describe each step in the cascade, discuss targeted interventions that address weak points in the continuum, review domestic and international policies that help shape and direct HIV care strategies, and conclude with recommendations and future directions for HIV providers and policymakers. While we primarily examine issues related to domestic HIV care in the US, we also discuss international applications of the continuum in order to provide broader context.

## Review

### Introduction

The HIV care continuum is an internationally-recognized framework, initially depicted in the United States (US) to represent HIV care as a progression from testing to engagement in medical care, antiretroviral therapy (ART) treatment, and, ultimately, suppression of the virus [1–4]. Although the terms “cascade” and “continuum” are often used interchangeably, in this review we use continuum to refer to dynamic and bidirectional navigation of the spectrum of HIV care engagement at an individual level, while treatment cascade examines a static, cross-sectional representation of these steps at a population level. The HIV care continuum gained considerable visibility in the US in 2013 due to the formation of the HIV care continuum initiative, created through Executive Order by the President, which prioritizes the establishment of a set of national indicators for HIV care [5]. These ten indicators, as detailed in the recently updated National HIV/AIDS Strategy (NHAS) for the US, reflect quantifiable 5-year goals for 2020 [6]. The updated NHAS synthesizes recommendations from the HIV care continuum initiative, incorporates findings from contemporary HIV/AIDS research, and highlights changes in HIV healthcare due to the passage of the Affordable Care Act (ACA) [6].

Although the Centers for Disease Control and Prevention (CDC) emphasize the importance of universal HIV testing at one end of the continuum and viral suppression at the other [1], researchers have drawn attention to the fact that steps in the middle of the continuum—linkage to care (LTC) and retention in care (RiC)—are vital in securing optimal health outcomes [3, 7, 8]. This is perhaps best exemplified in the national estimates of the number of persons living with HIV (PLWH) who fall at each end of the continuum: Whereas 86% of all PLWH in the US know that they are HIV-infected, an estimated 30% actually achieve viral suppression [1, 2]. The HIV treatment cascade is usually represented as a unidirectional series of five steps, including HIV diagnosis, LTC, engagement/retention in care, ART initiation and adherence, and viral suppression [1, 3, 7]. However, some researchers have suggested that the true nature of HIV care is often nonlinear, given that PLWH may iteratively transition in and out of treatment [3, 9, 10]. Moreover, a model developed by Gardner and colleagues suggests that an improvement in just one step of the treatment cascade would have little effect on the ultimate goal of viral suppression, underscoring how imperative it is to facilitate efficient and effective passage of PLWH through each step [2].

With continued advances in HIV treatment and increased knowledge regarding best practices for care, there is no justifiable reason that PLWH should receive suboptimal linkage to and retention in care. Yet, the majority of PLWH in the US do not have their disease adequately managed, and, every year, approximately 50,000 people are newly infected with HIV [11]. In this current issues review, we focus on successive steps of the HIV treatment cascade in which we provide summaries of each component, describe data monitoring strategies for accurately accounting for PLWH at each step, and discuss effective interventions specific to each phase. We also examine recent changes in US healthcare policy and how these changes interface with the HIV continuum, and we offer recommendations and future directions for HIV providers and policymakers. Although the focus of this review is the HIV care continuum as applied to PLWH in the US, we will also provide international context, as indicated.

### Diagnosis

Entry into the HIV treatment cascade begins with diagnosis. Current CDC guidelines recommend that healthcare professionals offer every patient between the ages of 13 and 64 an HIV test at least once, as an initiative to make HIV screening a routine rather than risk-based practice in healthcare settings [12]. HIV testing has never been more efficient or patient-friendly, as rapid test results can be available in under half an hour [13]; thus, integrating testing into routine health care visits is not a difficult task. Moreover, home testing is now available, providing individuals with enhanced access and control in learning their HIV serostatus. The US Preventative Services Task Force (USPSTF) has guidelines similar to the CDC’s for routine HIV testing [14], which has important implications for insurance coverage and payment for this screening test. However, it is estimated that approximately one in eight individuals who are HIV-positive are not aware of their serostatus, leaving ample room for improvements in domestic HIV testing and diagnosis [11]. This is not only a public health risk as it increases the probability that PLWH will unknowingly transmit HIV to others, but it is also detrimental to individual medical outcomes, as late diagnosis is associated with increased morbidity and mortality [15]. Further, Skarbinski and colleagues estimate that 91.5% of all new HIV infections are attributable to PLWH who are either undiagnosed or who are diagnosed but not retained in care [16]. In order to address these weaknesses in the continuum, one of the key goals set forth by the 2020 NHAS is to increase the number of PLWH who are aware of their status to 90% [6]. The Joint United Nations Programme on HIV/AIDS (UNAIDS) recently set international goals for 2020 termed the “90–90–90 target,” which aim to increase the number of PLWH who know their serostatus to 90%, and, among those who are diagnosed, to increase the percentage who are prescribed ART to 90% [17]. Likewise, among PLWH who are both diagnosed and on ART, the 90–90–90 target aims for 90% of PLWH to achieve and maintain viral suppression [17]. In response to the 90–90–90 target initiative, five cities shared their continuum of care data at the 21st International AIDS Conference, held in Durban, South Africa on July 18–22, 2016. [18]. These cities included San Francisco, California; Denver, Colorado; Amsterdam, Netherlands; Paris, France, and Kyiv, Ukraine, all of which have joined the global Fast-Track Cities Initiative that seeks to fulfill UNAIDS targets in urban areas with high HIV burdens [18]. In these fast-track cities, the percentage of PLWH who know their serostatus ranges from 51% in Kyiv to 93% in Amsterdam and San Francisco [18]. At the country and province levels, the US falls somewhere in the middle range, above British Columbia but below Denmark and Australia [19]. These numbers underscore the variability that exists even at the entry point of the continuum.

For low-income individuals in the US, Medicaid is their most common source of health coverage. As originally written, the ACA would have made routine HIV testing a universally-covered preventative screening measure; however, unequal Medicaid expansion has led to state-level variation in reimbursable routine HIV testing [20]. Currently, just 34 states cover routine HIV testing, while 16 cover only “medically necessary” HIV testing, which is defined by the state [20]. Notably, the states that do not offer reimbursable routine HIV testing are predominantly the ones with the highest HIV infection rates, and they are overwhelmingly found in the South [6, 20, 21]. This coverage disparity may bar individuals from getting tested, and it ultimately prevents PLWH who are unaware of their serostatus from receiving treatment.

### Linkage to care

Linkage to care (LTC) is the second step in the HIV care continuum. The CDC and Institute of Medicine (IOM) both define LTC as a period of three months or less between documentation of diagnosis and initiation of medical treatment with an HIV care provider/prescriber [1, 22]. The virtue of using a standard indicator is that it enables coordination among data tracking systems and facilitates policy analysis [22]; however, state reporting practices for reporting HIV diagnosis in the US vary, making it difficult to establish a standard baseline for measurement purposes [23, 24]. It is even more difficult to compare LTC rates among countries because there are multiple statistical “back calculation” methods used to estimate the number of undiagnosed PLWH. [25]. According to a recent systematic review of the literature, outside of the US, only the countries of Georgia, Denmark, and Australia and the Canadian province of British Columbia have population data at all steps of the continuum. [25].

Although viral suppression is achieved more quickly if ART is started within three months of diagnosis [26], estimates of the proportion of PLWH who are linked to care within 3 months in the US are between 59% [2] and 80% [1]. In light of low LTC estimates, the updated NHAS has set a 2020 goal of 85% of PLWH who are linked to care within 1 month of diagnosis, rather than 3 months, an ambitious target [6]. In other high-income countries and provinces, LTC proportions are higher, although none reach the targeted 85%. In British Columbia, between 61 and 67% of PLWH who are aware of their serostatus are linked to care, while estimates of LTC percentages in Denmark and Australia are higher, at 78% of PLWH in Australia and 81% in Denmark [19, 27]. This exemplifies the fact that even in high-income regions with universal health care systems, like British Columbia and Denmark, many PLWH drop out of the continuum before they have even received care. In low-income countries LTC proportions are appreciably lower, although estimates are derived from targeted intervention data rather than population-based data. In these countries, it is common for PLWH to delay treatment for several years until the disease has rapidly progressed [28]. For example, the proportion of PLWH linked to care in Kenya is 42% [29] and in South Africa, 37% [30].

Several multi-site studies have developed strategies to facilitate LTC. For example, although created for the purpose of testing and linking drug users to HIV care, the seek, test, treat, and retain (STTR) data collection and harmonization initiative is an HIV care model that is broadly applicable to many vulnerable populations [31]. As the authors discuss, what makes this model successful for HIV care is that it is built around a specific subgroup (e.g., drug users) and involves the collaboration of an interdisciplinary, multi-site team. Further, data interoperability is ensured through the use of standardized surveys and questionnaires which collect data on a number of measures, such as drug and alcohol use, mental health, and HIV testing history [31]. By streamlining the strategy’s approach and standardizing data collection and measurement techniques, the STTR Initiative facilitates coordinated research that, in turn, helps identify at-risk populations and link them to care [31]. Similarly, the multi-site access to care (A2C) initiative was developed for a specific subgroup—those living in poverty—and it expands on the HIV care continuum, primarily LTC [32]. In addition to using a set of quantifiable indicators, A2C also collects qualitative case studies to fully capture the experiences of staff members at each program site [32]. Further, A2C includes a cost-analysis component, which is especially useful for sites who may not have many resources. The STTR and A2C programs exemplify how LTC strategies may be developed in such a way that they address the needs of the target population, while also maximizing the resources available to the provider site(s).

### Retention

There is a lack of consensus about how to best measure retention, or continuity, in HIV care. While the IOM, NHAS, and CDC define HIV retention in care (RiC) as the proportion of PLWH who have “two or more visits for routine HIV medical care in the preceding 12 months at least three months apart” [22], these measures only account for adherence to scheduled medical appointments and not for missed or cancelled appointments [3]. Thus, it is recommended that any measure of RiC include at least two indicators—one for kept appointments and one for missed appointments—as they appear to provide complementary information [3, 33]. In fact, the number of missed appointments is a significant predictor in measuring clinical outcomes. Several studies have found that the number of missed medical appointments is significantly associated with higher viral loads and lower CD4 counts [4, 34]. Moreover, national racial disparities in HIV health outcomes [24, 35] may actually belie the presence of other factors. For example, in a multi-site trial, Zinski and colleagues found that the lower proportion of viral suppression found among African Americans in comparison to other racial groups loses statistical significance when accounting for the number of missed visits [36].

Compared to other high-income areas of the world, the US has the lowest proportion of PLWH who are retained in care. In British Columbia, 57% of diagnosed PLWH are retained in care [19, 27], and 75 and 76% are retained in Denmark and Australia, respectively [19]. In the US, the CDC (2014) approximates that just 40% of diagnosed PLWH are retained in care [1]. However, Yehia and colleagues suggest that this estimate is too low because it only measures the number of kept clinic visits over a single year, which neither captures longitudinal trends in accessing HIV care nor measures missed clinic appointments [37]. In reality, PLWH may transition in and out of care over time, referred to as “churning,” increasing the error margin of a point estimate [3, 4, 37]. Moreover, PLWH may be inaccurately designated as “out of care” due to undocumented changes in their health provider, incarceration, or death [3]. For example, the results of a San Francisco-based HIV surveillance project found that more than half of the patients who qualified as disengaged from care in local health records had merely changed providers [9]. This further illustrates the need for coordination among service providers so that data reporting can be timely and accurate. This is particularly challenging and pertinent for measuring the longitudinal estimate of HIV RiC, which is iteratively more complex than the dichotomous steps of testing and LTC.

### ART adherence

Although an International Association of Physicians in AIDS Care (IAPAC) expert committee recommends that health providers use patient self-report as a measurement of ART adherence, there is no standard indicator for confirming that patients consistently stay on ART medication [7, 8, 38]. Thus, while estimates for the number of all PLWH (undiagnosed and diagnosed, regardless of care engagement) prescribed ART in the US range from 24 [2] to 37% [1], it is unknown how many PLWH are consistently adherent to prescribed regimens. Internationally, the US again performs poorly on this measure: adherence estimates in other high-income areas range from 44% in British Columbia [27] to 66% in Australia [19].

One difficulty with monitoring adherence is that it is challenging to measure, as it relies largely on patient self-report. To illustrate, the US Medical Monitoring Project (MMP) found that 86% of PLWH in medical care reported taking ART as prescribed over a period of 3 days [39]. However, this estimate offers just a snapshot of patients’ self-reported adherence patterns, rather than a longitudinal record, and must be interpreted with caution. ART adherence is vital, as it is the primary determinant of viral suppression, which reduces the chance that PLWH will transmit the virus by 96% and helps prevent the development of ART-resistant strains of the virus [8, 22, 38, 40].

While the standard HIV care model places ART adherence after the retention stage, Hallett and Eaton suggest that a unidirectional model fails to represent other entry points into a “leaky” continuum, wherein PLWH are lost at each step [41]. In fact, their model predicts that the number of PLWH who are at the ART Adherence stage is greater than the standard linear model would suggest [41]. To explain this contradiction, they suggest that the traditional continuum model does not reflect the experience of PLWH who may reengage in care after dropping out of for a period of time, or who may even wait until their symptoms have become unmanageable to begin ART [41]. Other researchers have similarly proposed that PLWH may seek care in an intermittent manner, rendering the traditional cascade model too simplistic as an individual level monitoring tool [3, 9, 10].

According to an expert panel of the International Association of Physicians.

in AIDS Care, physicians should prescribe ART to PLWH immediately after their HIV diagnosis, regardless of CD4 count [38]. This is because lower CD4 counts weaken the immune system and increase the risk of opportunistic infections, AIDS-related diseases, and even non-AIDS-related diseases caused by chronic inflammation [15, 38]. Unfortunately, some countries continue to set national threshold guidelines for physicians. In South Africa and in many Latin American countries, for example, ART prescription protocol dictates that CD4 counts must first reach a suboptimal level (e.g., less than 500 cells per milliliter) [28, 42]. While practices like these are often a function of limited resources and are intended to prioritize care to PLWH most in need, a recent cost-benefits analysis indicates that early ART prescription is actually more cost-effective, even in low-income countries where medical resources are limited [43].

### Viral suppression

The final step in the continuum is also the primary goal of HIV treatment and public health interventions—viral suppression through ART. The CDC considers PLWH virally suppressed if their most recent viral load, measured within the past year, is less than 200 copies per milliliter (c/mL) [1]. However, there are an estimated 70% of PLWH in the US [1] who are not virally suppressed, including both PLWH who are not aware of their infection status and PLWH who have been diagnosed but are not in care. Internationally, not all countries and provinces use the same cutoff for viral suppression, which makes comparison more difficult. However, when using a more restrictive 50 c/mL as the cutoff, viral suppression estimates range from 25% in the US to 62% in Australia [19]. Using this comparative method, the US once again ranks lowest with respect to HIV outcomes.

Yet, for PLWH who are diagnosed, retained in care, and adhering to ART—in other words, PLWH who have completed each step of the HIV care continuum—90% have achieved viral suppression in the US [35]. Recent studies have also demonstrated that simple, cross-sectional measures of viral suppression are prone to misclassification [44, 45]. As for RiC, viral suppression is not constant once achieved, and patients often transition between suppressed and non-suppressed states, even over periods as short as 1 year.

The updated NHAS has set a goal for increasing the proportion of viral suppression among diagnosed PLWH to 80% by 2020 [6]. In order to achieve this target, HIV providers must work to link and retain PLWH in care. Beyond individual health benefits, success in this step is vital to success of a test-and-treat strategy, or “treatment as prevention” method, and is vital in preventing additional infections [2, 8, 45]. This method has proved effective even in rural African countries, where one community-based HIV testing and counseling intervention resulted in a 77% viral suppression rate for participants who were retained for a measured one-year period [46].

### Healthcare policy and access across the continuum

While many PLWH in the US have become eligible for health insurance as a result of the ACA, some HIV providers have expressed concern that patients will fall out of care, especially those who have previously received specialized services for PLWH through the Health Resources and Services Administration (HRSA)’s Ryan White HIV/AIDS Program (RWHAP) [47, 48]. First created in 1990 to address what was then termed the “AIDS crisis,” today the RWHAP serves over half a million PLWH who are either uninsured or underinsured, providing both supportive services (e.g., case management) and direct medical services (e.g., funding to medical clinics), including access to ART via the AIDS Drug Assistance Program [48]. Thus, while the ACA provides a tremendous source of new and additional coverage, RWHAP will remain a valuable “payer of last resort” for low income PLWH [47, 48], especially in states that have not expanded Medicaid to all childless, non-disabled adults under age 65 with incomes at or below 138% of the Federal Poverty Level [49]. In fact, data from the most recent RWHAP Annual Client-Level Data Report [47] indicates that a quarter of PLWH in the US still lack health insurance, highlighting the need for continued funding of the program. In light of the RWHAP’s significant role in providing HIV care to PLWH in the US, current literature urges HIV providers to advocate for continued funding of RWHAP [50–53], which has not been reauthorized since 2013 and relies on yearly appropriations from Congress [54].

At the diagnosis stage of the continuum, the US is on par with other high-income countries and provinces. However, the number of PLWH who are lost to care at the LTC stage subsequently positions the US behind other countries like Australia and Denmark, and the gap widens at each step. Ultimately, this should be a call-to-action for the US, which excels in HIV testing and diagnosis but falls behind other countries with regards to care. Until the US addresses all steps in the continuum of care, NHAS and UNAIDS goals for 90% viral suppression will be unattainable.

## Conclusions

The HIV treatment cascade is a straightforward, helpful guide for healthcare providers working with PLWH as it illustrates snapshot, population-level estimates of successive steps from diagnosis to viral suppression. Further, the IOM, NHAS, CDC, and UNAIDS movements toward standardization of national and international quantitative indicators of the cascade’s successive steps facilitates measurement, monitoring, and provision of HIV care. Yet, even with research-supported national guidelines, a large proportion of PLWH are lost from care at each step of the cascade in the US. In order to mitigate these losses, it is critical to address all steps in the HIV care continuum at an individual-level, most notably the linkage and retention stages which have received scant intervention attention [3]. Moreover, as emphasized in the 2020 NHAS, it is important to strengthen the data coordination infrastructure so that researchers and providers alike have access to instructive patient data that is systematic and comparable across geographic jurisdictions and over time. This is especially important given that many PLWH who gained health insurance through the ACA may have changed health providers, increasing the chance that data will be lost or incorrectly entered if organizations are not vigilant.

Through concerted collaboration, policymakers, HIV providers, and other key stakeholders have the ability to improve outcomes at each step of the HIV care cascade at a population level. The very fact that churning is such a frequent feature of HIV care illustrates the complexity of the treatment cascade and of the various individual, social, community, and public policy factors that shape PLWH’s ability to remain in care, and it highlights the importance of maintaining a socioecological perspective when addressing the needs of PLWH [55]. While there are various pathways to enter and progress through a more dynamic continuum at an individual level, the ultimate goal remains the same. Sustained viral suppression is the key to optimal health outcomes at both the individual and population levels, and treatment does serve as prevention. By strengthening outcomes at each step of the HIV care continuum, we can move closer to achieving the NHAS’ goal of “a future free of new HIV infections in the United States and healthier, longer lives for people living with HIV” [6]. This domestic goal begins with expanding free HIV testing services for people living in every state, in order to facilitate entry into the continuum and prevent inadvertent transmission. Further, as global comparison data show, the US must vigilantly address LTC strategies, as it is at this step in the continuum where the US falls behind other high-income regions of the world. For all countries, improving outcomes at each stage of the continuum aligns with UNAIDS’ “90–90–90 target” and central mission to facilitate a future AIDS-free world. Increasing the proportion of PLWH who remain engaged at each step of the treatment cascade will help make this goal possible.

## Abbreviations

PLWH: persons living with HIV
HIV: human immunodeficiency virus
US: United States
NHAS: national HIV/AIDS strategy
ACA: Affordable Care Act
ART: antiretroviral therapy
CDC: Centers for Disease Control and Prevention
USPSTF: US Preventative Services Task Force
IOM: Institute of Medicine
UNAIDS: Joint United Nations Programme on HIV/AIDS
LTC: linkage to care
STTR: seek, test, treat, and retain
A2C: access to care
RiC: retention in care
CD4: cluster of differentiation 4
IAPAC: International Association of Physicians in AIDS Care
MMP: Medical Monitoring Project
RWHAP: Ryan White HIV/AIDS Program
AIDS: acquired immune deficiency syndrome
c/mL: [Viral] copies per milliliter
